# Significant Suppression of Multiple Sclerosis in the Mouse EAE Model Using the PrC-210 Aminothiol

**DOI:** 10.3390/ijms262110597

**Published:** 2025-10-30

**Authors:** William E. Fahl, Bryan L. Fahl, Sarah R. Goesch, Hannah R. Goesch, Torsten R. Goesch

**Affiliations:** 1 Obvia Pharmaceuticals Ltd., Mountain View, CA 94043, USAsgoesch@stanford.edu (S.R.G.); hgoesch@stanford.edu (H.R.G.);; 2 Wisconsin Institutes for Medical Research, Department of Oncology, University of Wisconsin-Madison, Madison, WI 53705, USA

**Keywords:** dimethyl fumarate, DMF, Tecfidera, demyelination, gastric toxicity, leukopenia

## Abstract

Multiple sclerosis (MS) is a complex disease marked by chronic neuroinflammation and reactive oxygen species (ROS) toxicity in the central nervous system (CNS). Based on this ROS-driven mechanism, we tested whether PrC-210—a new aminothiol ROS scavenger—could lessen MS symptoms in mice with experimental autoimmune encephalomyelitis (EAE)-induced MS. Our goals were to assess the role of ROS in MS and evaluate the potential benefits of PrC-210 for managing MS. Mice with EAE received varying doses of PrC-210 under preventive and therapeutic protocols. Disease progression was measured using clinical scores and spinal cord histology. Safety was assessed by comparing the gastrointestinal and hematological toxicity between PrC-210 and dimethyl fumarate (DMF, Tecfidera’s active agent). PrC-210 reduced MS severity by up to 62% in paralysis scores versus those in the controls (*p* = 0.0001), whether used preventively or at the onset of paralysis. The group with the greatest decrease also showed the best spinal cord preservation and least demyelination. DMF caused toxicity at a dose that was ineffective, while PrC-210 showed no toxicity at effective levels. These findings suggest that the systemic administration of PrC-210 may offer a safe, effective MS treatment when started at symptom onset.

## 1. Introduction

MS is a chronic neuroinflammatory demyelinating disease of the CNS which is driven by an autoimmune response against CNS-specific myelin proteins that are produced by the oligodendrocytes (OLs) [1]. Various reports have highlighted key factors in the pathogenesis of MS, such as predisposing environmental and genetic causes, as well as inflammation and oxidative stress (i.e., a state of redox imbalance) [2]. OLs and their precursors, oligodendrocyte progenitor cells (OPCs), are highly susceptible to oxidative damage, as they possess a high intracellular iron concentration and high levels of Polyunsaturated Fatty Acids (PUFAs), which are highly susceptible to peroxidation. Furthermore, OLs have a high metabolic rate to enable the maintenance of myelin, as well as a lower endogenous free radical scavenging capacity as compared to that in the macrophages and astrocytes [3,4]. In MS, the pathological immune-mediated attack and associated oxidative stress in the OLs promote demyelination, axon degeneration, and neuronal dysfunction; this manifests as inflammatory focal lesions within the white matter of the brain, the optic nerve, and the spinal cord [5,6,7].

Oxidative stress has long been proposed to play a major role in the initiation and progression of MS pathology. Studies evaluating the degree of oxidative stress in MS patients have found increased levels of lipid peroxidation products (e.g., Malondialdehyde (MDA)) in their blood, cerebral spinal fluid, and urinary samples when compared to those in controls [8]. Analyses of MS lesions have also found increased levels of free-radical-producing enzymes, which have been linked to a progressive MS phenotype [8]. Furthermore, multiple MS animal studies, which have utilized the EAE model, have further explored the role of oxidative stress in MS. In these studies, increased activation of the Nrf2–antioxidant response element signaling pathway, which induces the expression of antioxidant genes, was able to markedly reduce MS disease progression [2]. Conversely, Nrf2-deficient mice have been found to harbor an exacerbated form of EAE MS pathology, showing increased immune cell infiltration, gliosis, and neuroinflammation [9].

Besides the role of oxidative stress, there have been two prevailing theories that have attempted to explain MS pathogenesis: the Outside-In theory and the Inside-Out theory. According to the Outside-In theory, environmental triggers, for example infections (e.g., Epstein–Barr virus), activate peripheral immune cells, including CD4+ and CD8+ T cells, B cells, and monocytes. Through mechanisms such as molecular mimicry (with foreign antigens resembling self-antigens) and bystander activation, autoreactive T cells are thought to mount an autoimmune attack against CNS-resident OL antigens (e.g., Myelin Basic Protein (MBP), Proteolipid Protein (PLP), and Myelin Oligodendrocyte Glycoprotein (MOG)) by crossing the blood–brain barrier (BBB) [10].

By comparison, the Inside-Out theory proposes that OLs may die due to intrinsic factors such as metabolic stress, mitochondrial dysfunction, or genetic predisposition, resulting in the release of normally sequestered CNS antigens into the extracellular space. As a result, primary CNS resident immune cells become activated and further propagate the destruction of OLs and neurons, further releasing more autoantigens (e.g., Myelin Oligodendrocyte Glycoprotein (MOG)) and eliciting a secondary immune response in the peripheral immune cells [11,12].

The release of antigens by degenerating OLs and neurons is recognized by CNS-resident immune cells (e.g., microglia and astrocytes) as a Damage-Associated Molecular Pattern (DAMP) via their Pattern Recognition Receptors (PRRs); this results in the induction of an inflammatory cascade [13]. Microglia, as the main antigen-presenting cells (APCs) in the brain, in addition to perivascular macrophages and dendritic cells, upregulate their MHC class I and II molecules, which enables the presentation of autoantigens to peripheral autoreactive CD4+ and CD8+ T cells, which promotes their activation and clonal expansion. Additionally, the microglia release a surge of proinflammatory mediators (e.g., TNFα, IL-1a, IL-1β, IL-18, C1q), which further promote immune cell recruitment, astrocyte activation, and amplification of the inflammatory cascade within the CNS [14,15,16,17].

Under pathological neuroinflammatory conditions, glial cells (i.e., microglia and astrocytes) are well known for expressing ROS- and reactive nitrogen species (RNS)-generating enzymes, which bombard the surrounding cellular milieu with ROS. NADPH oxidases (e.g., NOX2, NOX4) play a vital role in this toxic process because of their ability to catalyze the formation of superoxide (O_2_^•−^). This highly reactive free radical can either react with close-by structures or undergo dismutation (either spontaneously or catalyzed by superoxide dismutase (SOD)) to generate the more stable hydrogen peroxide radical (H_2_O_2_). Hydrogen peroxide can penetrate cell membranes, enabling the formation of highly reactive hydroxyl radicals (∙OH) in the presence of intracellular iron (i.e., the Fenton reaction), which can cause oxidative damage to various biological components (e.g., lipids, DNA, proteins). In a similar manner, glial cells also upregulate the expression of cytosolic inducible nitric oxide synthases (iNOS), which can react in vast amounts with superoxide to form the highly oxidizing peroxynitrite (ONOO^−^) [18,19,20,21].

We hypothesize that elevated levels of free radicals directly contribute to the initiation and accelerated progression of MS. Consequently, we expect that the systemic, interventional administration of a direct-acting free radical scavenger will attenuate MS disease progression.

**Figure 1:**
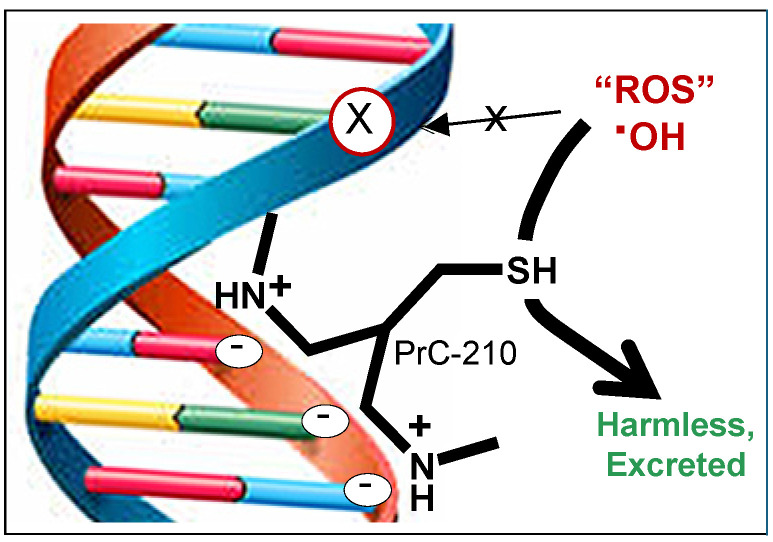
An experimental schematic showing PrC-210’s basic mechanism of action. Its (+) charged amines at the cellular pH of 7.2 “hover” through ionic interaction with the (−) charges that cover the deoxyribose DNA backbone. The ROS scavenging thiol group is projected three bond lengths away from the DNA helix into the nuclear milieu to scavenge ROS before it attacks dG residues in DNA (visualized by the X on the DNA backbone).

Free radicals are increasingly implicated in the pathophysiology of MS, yet their role in disease onset and progression remains incompletely understood. In this study, we proposed administering PrC-210 to experimental MS mice to efficiently scavenge ROS and determine whether this affected MS disease severity. PrC-210 is recognized as the most effective ROS scavenger currently known, consistently surpassing conventional antioxidants by restoring oxidative injury markers to their baseline values and demonstrating superior efficacy as a free radical scavenger (Figure 1) [22,23,24,25,26]. PrC-210 is a novel aminothiol ROS scavenger that can be administered orally, intravenously, and topically with no measurable limiting side effects [27]. By quantifying the effect(s) of efficiently, and continuously, scavenging ROS in EAE model MS mice, we hoped to both (i) elucidate the role(s) of elevated free radical levels in MS initiation and progression and (ii) demonstrate the potential efficacy that PrC-210 can achieve in clinical MS settings.

## 2. Results

### 2.1. Incidence and Initial Prevention of EAE Paralysis

**Figure 2:**
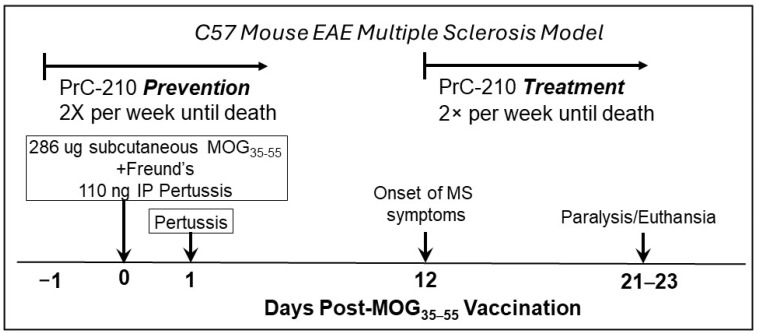
An experimental schematic illustrating the MOG_35−55_ peptide, Freund’s adjuvant, and Pertussis Toxin vaccination schedule to initiate EAE pathology in C57 mice. Mice were examined and scored daily through the 23-day experiment timeline; there were 10−12 mice per treatment group. EAE “clinical scores”, ranging from tail tip paralysis to severe limb paralysis, were assigned based upon the scoring key provided in ref. [28]. IP (intraperitoneal) injections of PrC-210 at the indicated fractions of the IP MTD (Maximum Tolerated Dose, i.e., a dose that would confer death to 1% of the injected mice) were administered twice weekly until mice were euthanized. Vehicle control mice received IP saline. PrC-210 was either administered in a “prevention protocol”, which began one day before MOG vaccination, or in a “treatment protocol”, which began the same day on which the first mouse in any group demonstrated tail tip paralysis. Mice were euthanized on the indicated days due to severe paralysis.

Vaccination of C57BL/6 mice with the MOG peptide followed by Freund’s adjuvant and Pertussis Toxin boosts (Figure 2 schematic) resulted in the onset of progressive paralysis on or around days 10–12, with initial tail tip paralysis, which then progressed into the rear limbs and eventually into the front limbs. Mice were scored daily using the industry-accepted scoring protocol [28]. Mice were euthanized when paralysis was sufficiently debilitating to preclude ready access to food pellets and/or gelatinized water placed on the cage bedding.

**Figure 3:**
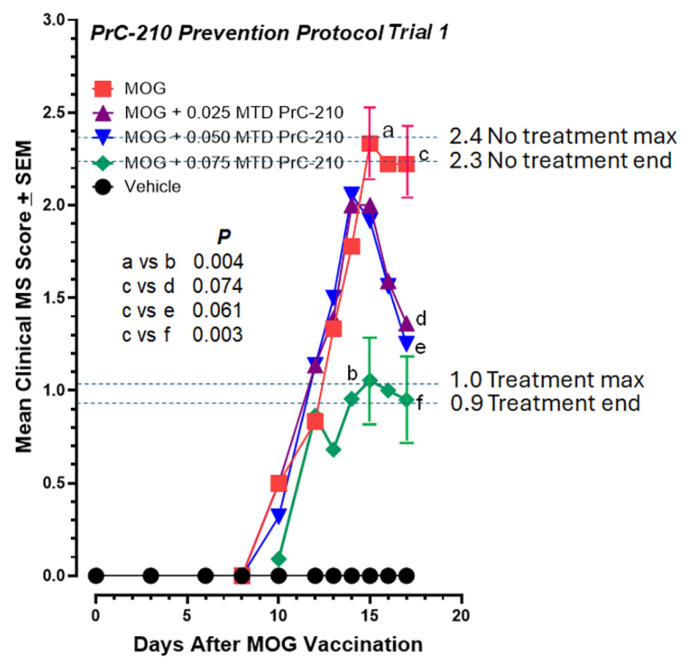
In the initial PrC-210 prevention protocol (see Figure 2 timeline), PrC-210 was administered twice weekly to the mice, with the first dose administered 1 day before the MOG peptide vaccination. PrC-210 doses are shown as fractions of the PrC-210 IP MTD, previously determined in C57 mice. Significant dose-dependent suppression of the EAE clinical scores (up to *p* = 0.003) was observed in the PrC-210-treated mouse groups. There was an N of 12 mice in each treatment group. Error bars indicate the SEM values at that data point. To determine statistical differences between the mean ± SEM on the indicated single days in the XY score plots, the 12 clinical scores from each point were pasted into a GraphPad “column analysis” format so that the column mean and SEM values could be compared using Student’s *t* test. *p* values are shown. “a–f” markers identify data points subjected to statistical comparison.

In this first test to determine whether systemic PrC-210 administration would suppress the severity of EAE paralysis, the first intraperitoneal (IP) injections of PrC-210 at three different doses, 0.025, 0.050, or 0.075 fractions of the PrC-210 IP Maximum Tolerated Dose (MTD; the mouse MTD for IP PrC-210 injection is 504 mg/kg body weight; the MTD essentially equals a dose lethal to 1% of the injected mice), were administered one day before the MOG vaccination (Figure 2). These doses were then continued twice per week until euthanasia. The specific doses above were chosen based on earlier ALS studies in our lab, which were able to suppress ALS pathology in ALS SOD^G93A^ transgenic mice in a twice-weekly well-tolerated dosing regimen. Whether compared at the “peak” paralysis scores (Figure 3) or at the “end” paralysis scores, the mice treated with 0.075 MTD PrC-210 showed a profound 60% suppression of paralysis (*p* = 0.003, 0.004). Because of the large standard deviations in the group scores, which are common in the EAE system, PrC-210 doses of 0.025 or 0.050 did not produce a statistically significant score reduction.

### 2.2. Initial Treatment Regimen to Suppress EAE Paralysis

**Figure 4:**
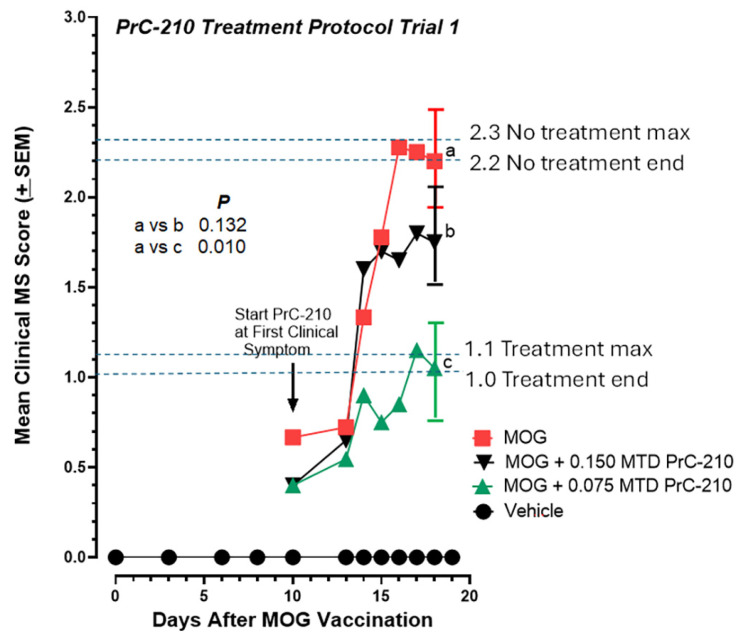
In the initial PrC-210 treatment protocol (see Figure 2 timeline), PrC-210 was administered twice weekly to mice, with the first dose administered on the same day in which a paralysis score was recorded in any group that had been vaccinated with the MOG peptide. PrC-210 doses are shown as fractions of the PrC-210 IP MTD. Significant dose-dependent suppression of the EAE clinical scores was observed in the two PrC-210-treated mouse groups. There was an N of 12 mice in each treatment group. *p* values for comparisons between treatment groups are shown. “a–c” markers identify data points subjected to statistical comparison.

Because the risk factors for MS disease development and its time of onset in human populations are not clearly defined, we sought to determine what effect, if any, systemic PrC-210 administration would have in a biweekly “treatment” regimen in which the first PrC-210 IP injection was begun on day 10 after one or more of the vaccinated mice demonstrated tail tip paralysis (Figure 4). When comparing the end paralysis scores, mice that received 0.075 MTD PrC-210 from day 10 forward showed a sizable 45% suppression of paralysis (*p* = 0.010) when compared to that in the vehicle controls. Mice that received twice that dose level, 0.150 MTD, showed a lesser response.

### 2.3. Higher-Dose PrC-210 Prevention Protocol to Suppress EAE Paralysis

**Figure 5:**
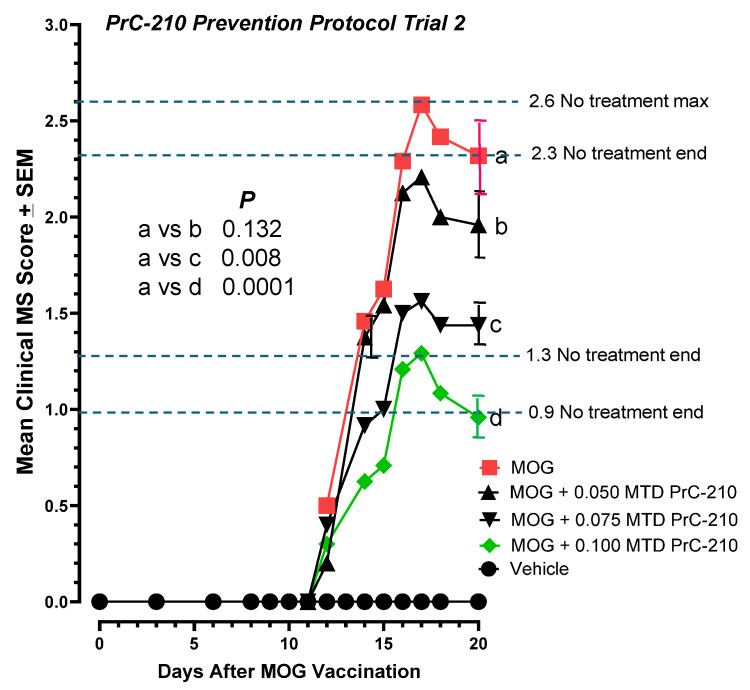
In the second PrC-210 prevention protocol, conducted months after Trial 1, including a higher-PrC-210-dose group (0.10 MTD), PrC-210 was administered twice weekly to mice, with the first dose administered 1 day before the MOG peptide vaccination. PrC-210 doses, as fractions of the IP MTD, are shown. Significant dose-dependent suppression of the EAE clinical scores (up to *p* = 0.0001) was observed in the PrC-210-treated mouse groups. There was an N of 12 mice in each treatment group. Error bars indicate SEM values at that data point. *p* values for comparisons between treatment groups are shown. “a–d” markers identify data points subjected to statistical comparison.

As was performed in the earlier PrC-210 “prevention” protocol, the C57 mice here received the IP drug twice weekly starting one day before the MOG vaccination (see Figure 2). Because the highest dose tested in the first prevention experiment (0.075 MTD) yielded the best protection, we added a higher PrC-210 dose here (0.100 MTD) to see whether it would work the same or better (Figure 5). The 0.075 MTD group protection efficacy here (*p* = 0.008) was very similar to that seen in the initial prevention protocol used months earlier (Figure 3). The 0.100 MTD PrC-210 group here yielded a substantial 60% suppression (*p* = 0.0001) of paralysis.

### 2.4. Higher-Dose PrC-210 Treatment Protocol to Suppress EAE Paralysis

**Figure 6:**
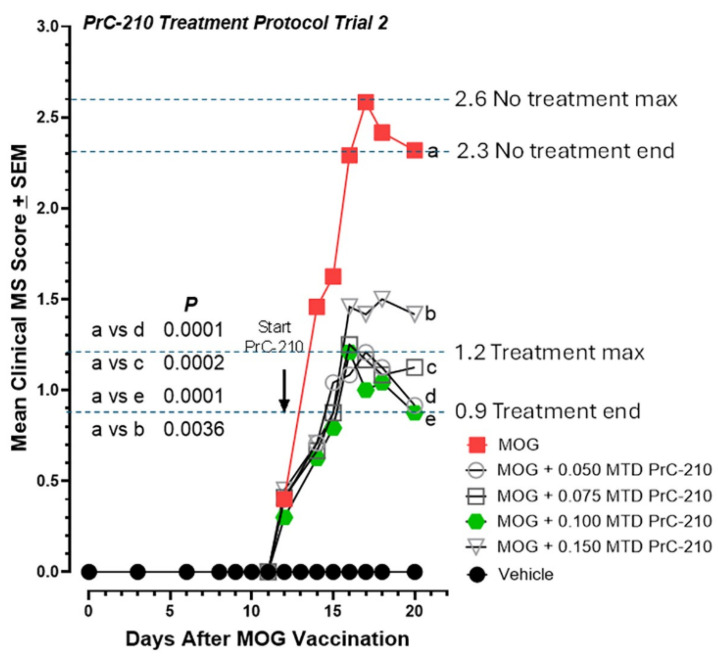
In a second PrC-210 treatment protocol, including two additional PrC-210 dose groups (0.05 and 0.10 MTD), PrC-210 was administered twice weekly to mice, with the first dose administered on the same day in which a paralysis score was recorded in any group that had been vaccinated with the MOG peptide. PrC-210 doses are shown as fractions of the PrC-210 IP MTD. Significant dose-dependent suppression of the EAE clinical scores was observed in these PrC-210-treated mouse groups (up to *p* = 0.0001 in two groups). There was an N of 12 mice in each treatment group. *p* values for comparisons between treatment groups are shown. “a–e” markers identify data points subjected to statistical comparison.

In this larger treatment study, groups of C57 mice were injected starting on day 12 after MOG vaccination with either 0.050, 0.075, 0.100, or 0.150 MTD PrC-210, twice weekly. Clearly, PrC-210 treatment of these mice as they actively develop substantial EAE-induced paralysis has a substantial effect in suppressing the severity of that process (Figure 6). All of the PrC-210 dose groups showed a significantly suppressed severity of developed paralysis. The 62% paralysis suppression seen in the 0.100 MTD group (*p* = 0.0001) was the largest effect seen, but all of the PrC-210 groups showed substantial efficacy, even when they were first administered it starting on day 12 post-MOG vaccination.

**Figure 7:**
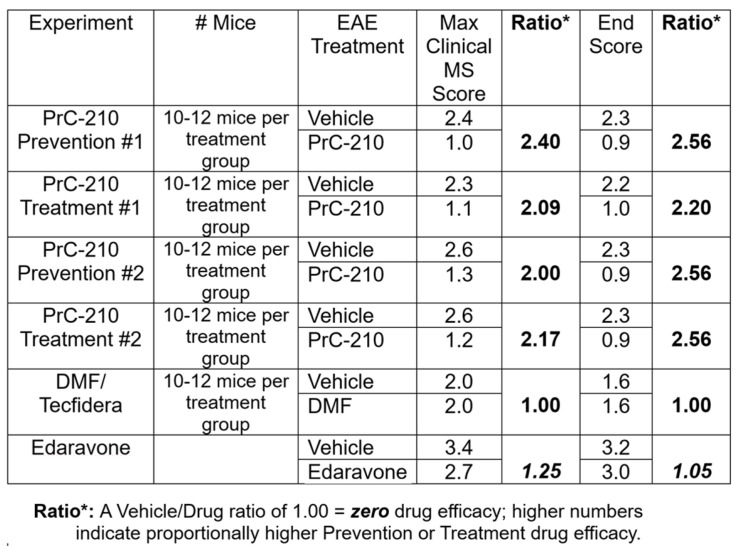
This table summarizes the substantial efficacy that PrC-210 shows in suppressing EAE clinical paralysis, whether administered in a prevention or treatment regimen to the MOG-vaccinated C57 mice. See reference [29] for data regarding Edaravone efficacy.

Figure 7 provides a summary of the efficacies seen in the above PrC-210 “prevention” and “treatment” regimens, as well as a comparison to (i) the absence of efficacy for DMF/Tecfidera in our study and (ii) the very modest score suppression shown in a published study on Edaravone’s efficacy in this mouse EAE model.

### 2.5. Spinal Histology Supports Paralysis Suppression Induced by PrC-210

**Figure 8:**
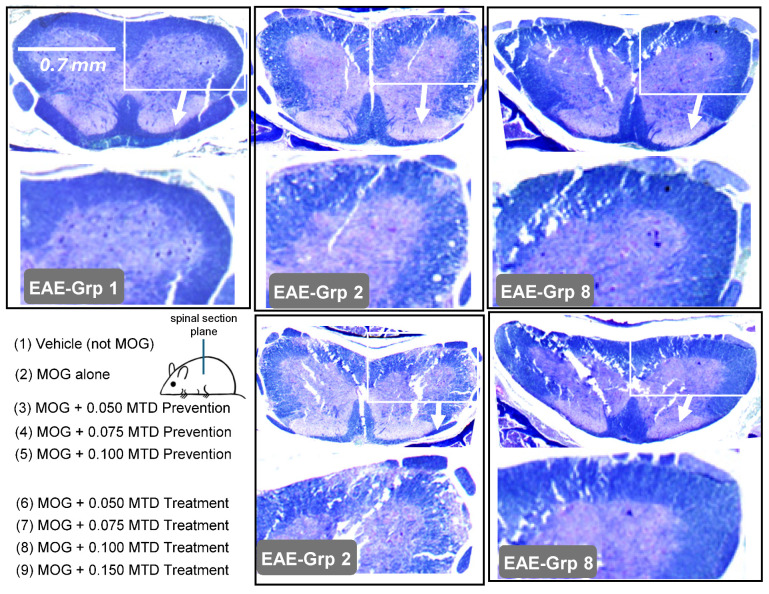
Spinal columns and ~0.5 cm of tissue flanking the spinal columns, from the neck to the tail base, were cut out with scissors from euthanized mice from each treatment group and fixed in formalin. Following de-calcification, fixed tissues were embedded into paraffin and sectioned in the plane shown in this figure’s inset of the mouse, and samples were then stained with Luxol Fast Blue to highlight myelin density and uniformity. For each spine image, the lower panel is a 3× enlargement of the spine cross-section to illuminate peripheral spine demyelination better in the MOG-vaccinated mice, and its substantial suppression by PrC-210, in both the prevention and treatment protocol groups.

On day 20 post-MOG vaccination, 2–3 mice from both the vehicle control group and each PrC-210 treatment group were euthanized, and their spinal column was removed en bloc, formalin-fixed, de-calcified, sectioned, and stained with H&E or Luxol Fast Blue to preferentially highlight myelin integrity in the spine cross-sections (Figure 8). The myelin integrity in the EAE-Grp 1 vehicle controls (with no MOG vaccination) is apparent, both in the low-power complete spine cross-section and the 3× magnification of the periphery of the spinal column.

The spine cross-sections from the two mice from EAE-Grp 2, which received MOG vaccination without treatment, show readily apparent demyelination and structural destruction of the spinal column periphery, completely consistent with the widespread rear and front limb paralysis seen in these Grp 2 mice.

The spine cross-sections from two Grp 8 mice (0.100 MTD) showed intact spinal integrity with little or no evidence of demyelination. This correlates extremely well with the 62% suppression of the EAE paralysis scores seen in Grp 8 (Figure 6), containing mice which received biweekly injections of 0.100 of the MTD of PrC-210.

### 2.6. Oral DMF’s Toxicity Versus That of Oral Control or Oral PrC-210 Treatments

#### Stomach Tissue Toxicity

Gastrointestinal toxicities have been commonly reported in humans who receive long-term administration of Tecfidera/DMF [30,31]. Following 21 days of daily, oral gavage treatment with 15 mg/kg of DMF, a regimen commonly used in the published literature on DMF and Tecfidera in the mouse EAE model [29,32,33], the mice were euthanized, and rinsed stomach tissue was examined using both gross white-light photography of the stomach tissue and fixation and then H&E staining and microscopic analysis of the gastric wall.

**Figure 9:**
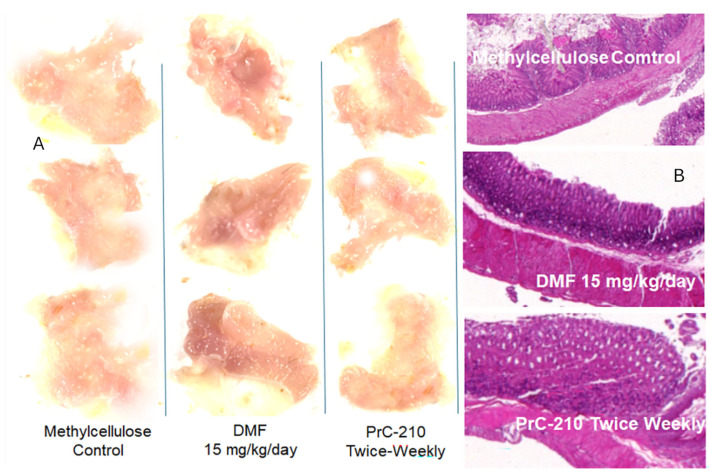
(**A**) White-light photographs of gross stomachs from mice after 21-day oral gavage treatment with either (i) 0.08% methylcellulose (vehicle control), (ii) 0.08% methylcellulose containing 1.5 mg/mL DMF, or (iii) 0.1 the MTD (180 mg/kg) of PrC-210 biweekly. (**B**) Histology sections (H&E staining) of the stomach walls from the stomachs shown in panel (**A**) (10× objective).

Each white-light image of the stomach wall was gathered into a single PowerPoint field image, and the collection of images were then equally “lightened” by +20%. Figure 9A shows that there was significant erythema throughout the DMF-exposed mouse stomachs. There was no detectable erythema in either the methyl cellulose controls or in the mice given the twice-weekly 0.1 MTD PrC-210 treatment, which conferred the maximum suppression of the EAE clinical scores, as shown in Figure 3, Figure 4, Figure 5, Figure 6, Figure 7 and Figure 8. Analysis of histology sections of the gastric walls (Figure 9B) reinforced the white-light image analysis. There was significant erosion of the gastric mucosa in the DMF-treated mice; this was accompanied by significant infiltration of the mucosa by basophilic inflammatory cells. This histological basophilia, not seen in the control or PrC-210-treated mice, explains the deeper red-blue coloration of the DMF-treated stomachs under white light (Figure 9A).

**Figure 10:**
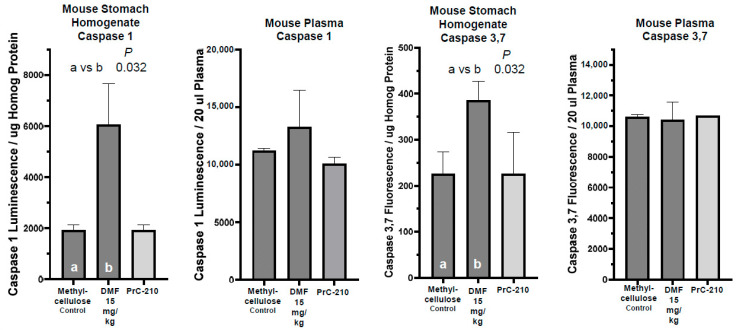
Measurement of caspase 1 inflammatory marker or caspase 3/7 cell death marker in homogenates prepared from stomach walls of the stomachs shown in Figure 9A or in blood plasma from the mice that provided the stomachs after the 21-day oral gavage treatments. Caspase marker levels were measured as described in Materials and Methods (Section 4.4.1, Section 4.4.2 and Section 4.4.3). *n* = 4 in each treatment group. “a” and “b” are indicators to allow for statistical comparison of columns.

Both stomach wall homogenates and blood and plasma from each DMF-treated group of mice were analyzed in vitro to determine whether elevated levels of either or both inflammatory and cell death biomarkers were found to be associated with the stomach wall erythema seen in the DMF-treated mice. The caspase 1 “inflammasome” levels in the DMF-treated stomachs was significantly elevated (*p* = 0.032), with a slight increase in plasma caspase 1 activity (Figure 10). Interestingly, the caspase 3/7 cell death marker was also significantly elevated in the DMF-treated stomachs (*p* = 0.032), with no detectable elevation in plasma caspase 3/7 levels.

**Figure 11:**
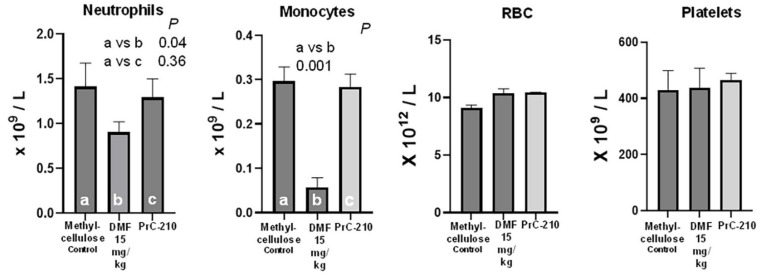
Levels of select blood cell populations in blood recovered from mice after 21-day oral gavage treatment with either methylcellulose control, methylcellulose containing 1.5 mg/mL of DMF, or biweekly PrC-210. Complete blood counts were made using an Abaxis HM% analyzer. “a”, “b”, and “c” are indicators to allow for statistical comparison of the columns.

Studies in the literature have reported “leukopenia” in mice treated with DMF/Tecfidera [31], so we performed complete blood counts in whole blood recovered from each mouse in this toxicity study. In Figure 11, we see that the 21-day DMF treatment was associated with a significant decrease in neutrophils (*p* = 0.04) and a profound decrease in monocytes (*p* = 0.001). There was no effect on the CBC endpoints in the PrC-210-treated mice.

### 2.7. Oral DMF’s Efficacy in Suppressing EAE Paralysis

**Figure 12:**
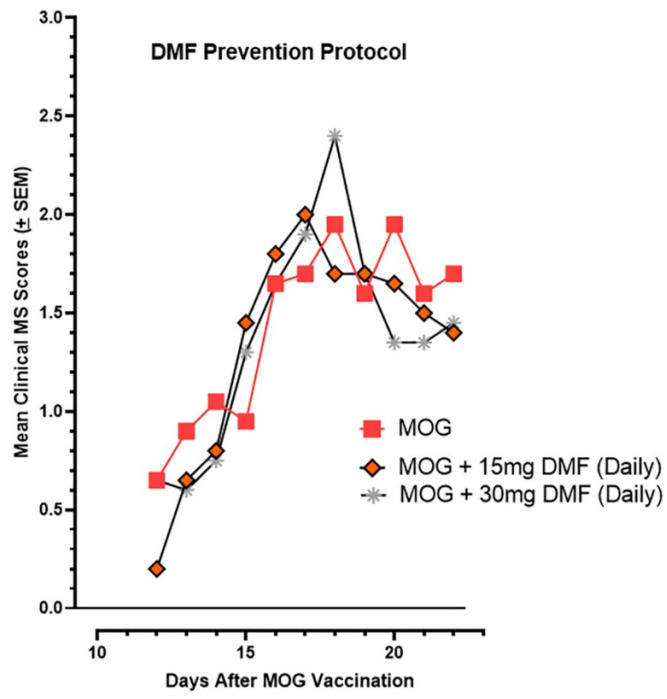
A 15 mg/kg oral gavage dose of DMF (in 0.08% methylcellulose:water) was administered daily to the MOG-vaccinated mice starting on the day of MOG vaccination and continued for 21 days. Control mice received the methylcellulose vehicle only. There was an N of 12 mice in each treatment group.

The twenty-one-day oral gavage treatment with 15 mg/kg/day or 30 mg/kg/day of DMF in the MOG-vaccinated mice showed no efficacy in terms of suppression of the paralysis endpoint (Figure 12).

## 3. Discussion

The search for both a clear mechanistic understanding of the etiology of MS in humans and its sustained, chronic debilitation in humans remains an ongoing challenge. Thoughtful, plausible explanations for both of the aforementioned pathomechanisms of MS are still continuously discussed, but both involve pathways that include chronic CNS neuroinflammation and associated ROS production and toxicity. In the context of this ROS-based mechanism, we asked whether the new PrC-210 aminothiol ROS scavenger (Figure 1), the best direct-acting ROS scavenger in biology when compared to the 13 other ROS scavenger/antioxidants most cited in PubMed [22,26], could effectively suppress the severity of EAE-induced MS in mice. Our results in this study showed the following: (i) PrC-210 was profoundly effective in suppressing MS severity (up to 62% suppression), whether administered in a “prevention” regimen or a “treatment” regimen, i.e., started at the onset of discernible MS paralysis. (ii) The best suppression of paralysis using PrC-210 was seen in the same group that showed the best histological evidence of PrC-210-associated integrity of the spinal structure and suppressed spinal demyelination. (iii) Comparison with DMF/Tecfidera (in Figure 7) shows that PrC-210 was 2.56 times (2.56/1.00) more effective in paralysis suppression than DMF/Tecfidera, and comparison to Edaravone shows that PrC-210 was 2.44 times (2.56/1.05) more effective in paralysis suppression than Edaravone. (iv) The ability of PrC-210 to efficiently scavenge and quench ROS, and its association here with 62% suppression of MS severity, clearly supports the basic mechanistic role that ROS toxicity has been assumed to play in human MS etiology, and (v) the 62% PrC-210 suppression of EAE-MS paralysis severity seen here in a “treatment” regimen implies that similar efficacy could be achieved in human MS patients.

In our study, we confirmed that oxidative stress is a major pathogenic mechanism in multiple sclerosis, contributing to neurodegeneration and increased clinical EAE severity. Importantly, we found that this detrimental effect can be counteracted through ROS scavenging, reinforcing the concept of oxidative stress as a modifiable therapeutic target. These findings align with the current scientific consensus and underscore that scavenging of ROS represents a potent intervention throughout the prevention, onset, and progression of the disease [8]. In this, the linearity between the dose-dependent reduction in ROS and clinical scores clearly implicates oxidative stress in centrally mediating the underlying disease pathology. Utilizing an efficacy score to measure the therapeutic efficacy of an agent, with scores at or lower than 1.0 representing no beneficial effect, we showed PrC-210 efficacy ratios of 2.65 and 2.44 when compared head to head, respectively, with DMF/Tecfidera and Edaravone. Our paralysis scores with vehicle treatment, in onset, severity, and duration, are consistent with previous findings from other groups using the EAE model.

This study also included a brief toxicology study to assess the relative toxicities between the DMF/Tecfidera active agents and PrC-210. The observed toxicities in mice, that received a dose of daily DMF (15 mg/kg) often used in the literature, closely replicated the gastric toxicity [30,31] and leukopenia [34] toxicities described extensively in the published literature. There were no gastric or blood cell toxicities observed for the PrC-210 dose that conferred the maximum suppression (i.e., 62%) of the EAE paralysis scores. Additional ongoing toxicology studies in swine likewise show the absence of any changes in the pigs’ CBC blood cell counts or 14 blood chemistry parameters in the 7 days following IV doses of PrC-210 at or above the determined NOAEL dose for pigs of PrC-210. The absence of PrC-210 toxicity, combined with 62% suppression of EAE paralysis, is a strong and compelling argument to carry PrC-210 forward into human MS clinical trials.

**Figure 13:**
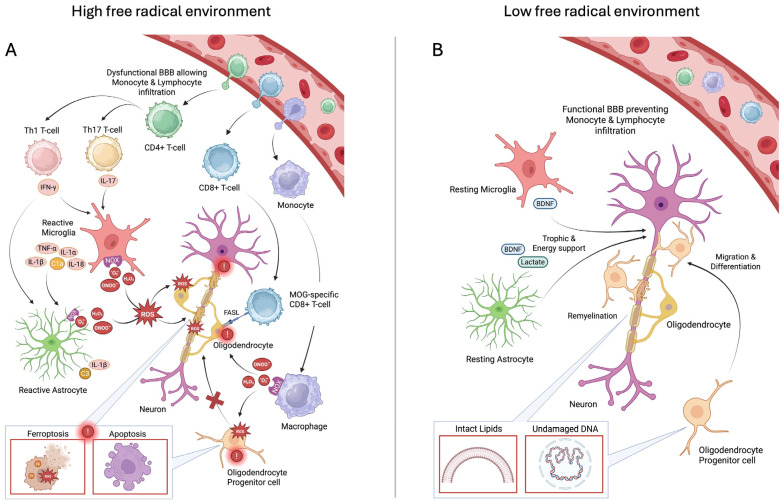
A hypothetical mechanistic schematic of how PrC-210 evokes its therapeutic efficacy in MS pathogenesis of the EAE model. (**A**) illustrates how the local generation of ROS causes cell death (depicted in red boxes above (e.g., Ferroptosis, Apoptosis)) in the OLs, neurons, and OPCs; induces neuroinflammation; and induces immune cell infiltration and activation in the CNS. The red X visualizes the inability to replace damaged OLs with OPCs, prohibiting remyelination. Exclamation marks indicate ROS-induced cell death. (**B**) illustrates how PrC-210’s efficient ROS scavenging properties (i) alleviate oxidative stress and thereby enable myelin regeneration through newly differentiated OLs, (ii) inhibit immune cell infiltration by restoring the BBB, (iii) suppress ROS-induced damage, and (iv) restore an anti-inflammatory local immune environment. Created with BioRender.com.

In Figure 13, we have assembled the proposed mechanistic outline to plausibly capture accepted elements of MS etiology and maintenance, as well as to plausibly explain how PrC-210 significantly reduces the degree and severity of the disease process. In the disease etiology (Figure 13A), activated microglia and macrophages recruit peripheral immune cells, such as lymphocytes, monocytes, and dendritic cells, to the CNS through chemokines; this results in their breaching of the BBB and their infiltration into the CNS parenchyma. Subsequently, autoreactive CD4+ and CD8+ T cells and monocytes are locally activated through proximal APCs (e.g., microglia, macrophages, dendritic cells) that present autoantigens and then release proinflammatory mediators (i.e., chemokines and cytokines), which promote their further differentiation. This initiated cascade of pathological, proinflammatory processes additionally promotes the upregulation of NADPH oxidases (e.g., NOX2, NOX4) and inducible nitric oxide synthases (iNOS) in the microglia, macrophages, and astrocytes. Increased levels of NOX-enzyme-generated superoxide radicals can promote cellular damage in close proximity, or farther way, if or when the superoxide radicals are dismutated into hydrogen peroxide. Because hydrogen peroxide is lipid-soluble, it can permeate cell membranes, and this then enables the generation of highly reactive hydroxyl radicals (∙OH) in the presence of intracellular iron (i.e., the Fenton reaction); this clearly facilitates oxidative damage to any cell components (e.g., lipids, DNA, proteins). An increased level of iNOS-catalyzed nitric oxide can similarly further react with superoxide to form highly oxidizing peroxynitrite (ONOO^−^). This surge of oxygen free radicals has severe detrimental effects on OLs and OPCs inducing cell death (e.g., intrinsic apoptosis (through DNA damage), ferroptosis (lipid peroxidation), axon injury (e.g., cytoskeletal elements, axonal mitochondria), and demyelination (e.g., lipid peroxidation of myelin sheaths)).

Past studies have shown that PrC-210 possesses the ability to neutralize a broad range of oxygen and nitrogen free radicals. Therefore, its direct mechanism of action, and efficacy, in the EAE MS model can plausibly be explained by its unequaled radical scavenging abilities and thus its ability to restore redox balance in cells.

Separately, numerous published studies have utilized the activation (or transgenic inactivation) of intrinsic free radical scavenging pathways or treatment to pharmacologically augment intrinsic radical scavenging (i.e., “antioxidants” which do not themselves scavenge radicals) and have shown modest diminutions in EAE severity [35,36]. In Figure 13B, we show the effects that logically follow from the introduction of the highly efficient, direct-acting (i.e., scavenging, per Figure 1) PrC-210 thiol into the CNS setting, where it (1) restores BBB integrity, preventing further peripheral immune cell infiltration; (2) reverses gliosis by shifting both the microglia and astrocytes into a resting state, which reduces microglial phagocytosis and the destruction of myelin, downregulates ROS-producing enzymes, and allows the astrocytes to provide trophic and energy support to the neurons; (3) enables OPCs to differentiate into OLs and restore axonal integrity; and (4) halts any further cell death in the OLs, neurons, and OPCs, thereby halting further progression of the pathology.

## 4. Materials and Methods

### 4.1. Materials and PrC-210

PrC-210 HCl (MW: 220) was synthesized for these studies as previously described [37]. C57BL/6 mice (female, 7 weeks age) were purchased from Jackson Labs (Bar Harbor, ME, USA). Solvents and chemicals were purchased from Sigma-Aldrich (St. Louis, MO, USA). EAE was induced using Hooke #EK-2110 Kits (Hooke Labs, Lawrence, MS, USA) [28]. Dimethyl fumarate (#242926) was from Sigma-Aldrich (St. Louis, MO, USA). Methyl cellulose (#M0512) was from Sigma-Aldrich.

### 4.2. Animal Studies

This research was approved by the School of Medicine and the Public Health Institutional Animal Care and Use Committee at the University of Wisconsin (Protocol #M006610). All procedures were performed in accordance with the Animal Care and Use Policies at the University of Wisconsin. Mice were maintained on a 12 h light/dark cycle and were provided water and lab chow ad libitum (Harlan Teklad 8604). The mice were acclimated in animal facility for seven days before MOG vaccination.

To induce EAE, on day 0, the mice received 2 × 100 μL SC (subcutaneous) injections of the prepared Hooke emulsion [28] containing both the MOG_35–55_ peptide (~285 μg total administered) and Freund’s adjuvant into their dorsal back and single IP (intraperitoneal) injections of Pertussis Toxin (110 ng/injection; Hooke Labs, above) on day 0 and day +2. As shown in the Figure 2 treatment schematic, in the “prevention protocol”, the mice received IP injections of the indicated doses of PrC-210 twice per week, starting on day −1, a day before the MOG vaccination). The IP MTD (Maximum Tolerated Dose) of PrC-210 was 524 mg/kg bw. As also shown in the Figure 2 treatment schematic, in the “treatment protocol”, the mice received IP injections of the indicated doses of PrC-210 twice per week, starting on the first day on which we observed a detectable EAE clinical score. Each mouse was scored daily for its EAE clinical score using the widely accepted criteria described in reference [28].

### 4.3. Histology

On day 20 post-vaccination, 2–3 mice per treatment group whose clinical scores best represented the group status on that day were euthanized, and their spinal columns and the surrounding tissue were dissected out en bloc and placed into 10% formalin. The 10-day-fixed tissue was first de-calcified, and the tissue was then embedded into paraffin, and 5-micron sections were then cut and mounted, de-paraffinized, and stained using Luxol Fast Blue or hematoxylin–eosin. Slides were scanned using a 10× objective on an Optika Optiscan10 scanner (Greenville, DE, USA) o generate jpg images, which were then analyzed.

### 4.4. DMF Toxicity Analyses

In a separate toxicity study conducted in non-MOG-vaccinated, wild-type C57BL/6 mice, either 0.08% methylcellulose in water as the control or 0.08% methylcellulose in water containing 1.5 mg/mL of DMF was administered via oral gavage once per day for 21 days, the normal duration of DMF treatment in MOG-vaccinated EAE mice [29,32,33]. The DMF mice received the 1.5 mg/mL DMF formulation at a daily gavage volume equal to 1% of their body weight, such as 240 μL in a 24.0 gm mouse. This equaled a daily dose of 15 mg/kg body weight to the mice. A separate group of mice received a twice-weekly 0.1 MTD gavage dose (180 mg/kg) of PrC-210, also in a water volume of 1% of the animal’s body weight. After 21 days of the daily treatments, the mice were euthanized. (i) Their blood was collected into an EDTA-containing tube, half of which was used for CBC determination and half of which was spun, and their plasma was frozen. (ii) Mouse stomachs were dissected out and then dissected open along the long, ventral surface of the stomach, laid open, pinned onto white Styrofoam, rinsed free of all food under a tap-water stream, (iii) photographed under bright white-light illumination, and (iv) then dissected into halves; one stomach half was immersed in 10% formalin for histology analysis (H&E staining), and one stomach half was placed into a tube and snap-frozen in liquid nitrogen for later homogenization and measurement of tissue inflammatory (caspase 1 Inflammasome) and cell death (caspase 3,7) biomarkers.

#### 4.4.1. Stomach Tissue Homogenization and Protein Determination

Frozen stomach tissue (from Section 2.4, above) was quickly thawed, 1.0 mL of caspase lysis buffer [38] was added to the tube, the tube was immersed in 4 °C ice water, and the tissue was homogenized for 30 s using a Polytron homogenizer (Thermofisher; Chicago, IL, USA) at full power. The tubes were placed on ice, and 5–10 min later, a 500 μL aliquot of homogenate was transferred into an Eppendorf tube, and the tubes were frozen at −80 °C prior to analysis. For the analyses, the tubes were thawed and placed on ice, and aliquots were used for protein determination with the Bradford reagent.

#### 4.4.2. Caspase 1 Assay

The activated caspase 1 activity in mouse stomach homogenate or 20 μL of mouse plasma was determined using the Caspase-Glo 1 Inflammasome Assay (#G9951, Promega, Madison, WI, USA). The activated caspase 1 assay was performed as follows: 20 µL of mouse plasma (stored at −80 °C) or stomach homogenate was mixed with 30 μL of buffer and 50 μL of the Z-WEHD caspase 1 substrate in the well of a white, opaque, 96-well plate. The plate was shaken at 200 rpm for 60 s and then allowed to sit at room temperature for 30 min. The chemiluminescence in the wells was measured using a BMG Clariostar luminescence plate reader. A caspase 1 internal standard was included in each experiment.

#### 4.4.3. Caspase 3, 7 Assay

The activated caspase 3,7 activity in mouse stomach homogenate or 20 μL of mouse plasma was determined using the Apo-ONE Caspase 3, 7Assay (#G7790, Promega, Madison, WI, USA). The activated caspase assay was performed as follows: 20 µL of mouse plasma (stored at −80 °C) or stomach homogenate was mixed with 30 μL of buffer and 50 μL of the undiluted Apo-ONE substrate in the well of a black, opaque, 96-well plate to initiate a 60 min reaction. Foil-covered plates were shaken at 200 rpm at 37 °C for 60 min. The DEVD caspase substrate peptide cleavage was measured using a BMG Clariostar fluorescent plate reader (Cary, NC, USA) at an excitation wavelength of 499 nm and an emission wavelength of 521 nm. A caspase 3/7 internal standard was included in each experiment to enable comparison between days.

#### 4.4.4. Mouse Blood Complete Blood Count (CBC)

Mouse blood that had been collected into EDTA-containing tubes was maintained at room temperature prior to CBC determination, performed using an Abaxis HM5 Hematology Analyzer (Abaxis, Union City, CA, USA).

#### 4.4.5. Statistical Analyses

For statistical analysis, Student’s *t* test was used for simple comparisons between groups. GraphPad Prism version 10.6.1 was used for analyses. There were no exclusions of animal group data.

## Data Availability

The original contributions presented in this study are included in the article. Further inquiries can be directed to the corresponding author(s).
